# Identification of HLA‐A*11:01‐restricted *Mycobacterium tuberculosis* CD8^+^ T cell epitopes

**DOI:** 10.1111/jcmm.12867

**Published:** 2016-04-12

**Authors:** Su‐Dong Liu, Jin Su, Shi‐Meng Zhang, Hai‐Ping Dong, Hui Wang, Wei Luo, Qian Wen, Jian‐Chun He, Xiao‐Fan Yang, Li Ma

**Affiliations:** ^1^ Institute of Molecular ImmunologySchool of BiotechnologySouthern Medical UniversityGuangzhouChina; ^2^ Department of Respiratory and Critical Care MedicineNanfang HospitalSouthern Medical UniversityGuangzhouChina; ^3^ Department of Severe Tuberculosis MedicineGuangzhou Chest HospitalGuangzhouChina

**Keywords:** MTB, CD8^+^ T cell epitope, class I restriction, ELISPOT, CFSE, Dextramer staining, active TB patient

## Abstract

New vaccines are needed to combat *Mycobacterium tuberculosis* (MTB) infections. The currently employed Bacillus Calmette‐Guérin vaccine is becoming ineffective, due in part to the emergence of multidrug‐resistant tuberculosis (MDR‐TB) strains and the reduced immune capacity in cases of HIV coinfection. CD8^+^ T cells play an important role in the protective immunity against MTB infections, and the identification of immunogenic CD8^+^ T cell epitopes specific for MTB is essential for the design of peptide‐based vaccines. To identify CD8^+^ T cell epitopes of MTB proteins, we screened a set of 94 MTB antigens for HLA class I A*11:01‐binding motifs. HLA‐A*11:01 is one of the most prevalent HLA molecules in Southeast Asians, and definition of T cell epitopes it can restrict would provide significant coverage for the Asian population. Peptides that bound with high affinity to purified HLA molecules were subsequently evaluated in functional assays to detect interferon‐γ release and CD8^+^ T cell proliferation in active pulmonary TB patients. We identified six novel epitopes, each derived from a unique MTB antigen, which were recognized by CD8^+^ T cells from active pulmonary TB patients. In addition, a significant level of epitope‐specific T cells could be detected *ex vivo* in peripheral blood mononuclear cells from active TB patients by an HLA‐A*11:01 dextramer carrying the peptide Rv3130c_194‐204_ (from the MTB triacylglycerol synthase Tgs1), which was the most frequently recognized epitope in our peptide library. In conclusion, this study identified six dominant CD8^+^ T cell epitopes that may be considered potential targets for subunit vaccines or diagnostic strategies against TB.

## Background

*Mycobacterium tuberculosis* (MTB) infects one‐third of the world's population with a majority of the cases occuring in developing countries. Of those infected, 5–10% are likely to develop active tuberculosis (TB) during their lifetime 1, 2. The World Health Organization estimates that in 2014 alone, 9.6 million people developed TB and 1.5 million died from it. Meanwhile, the prevalence of multidrug‐resistant TB and HIV coinfection increases the difficulty of preventing and treating TB 3.

Host–pathogen interactions between mycobacteria and humans are complicated and have not been fully elucidated. However, strong evidence suggests that adaptive CD4^+^ and CD8^+^ T cell responses represent an important mechanism for host recognition and contain of MTB 4. Major histocompatibility complex (MHC) class II‐restricted CD4^+^ T cells play an essential role in protective immunity against MTB; this is supported by evidence that HIV‐positive patients, who have reduced circulating CD4^+^ pools, are more susceptible to MTB infection 5. CD4^+^ T cells activate macrophages by releasing lymphokines such as interferon (IFN)‐γ and tumour necrosis factor‐α 6. CD8^+^ T cells protect against MTB by lysing infected cells and killing intracellular bacterial by the release of the antimicrobial peptide granulysin 7. β2‐microglobulin (β2m)‐deficient mice, which are unable to develop MHC class I‐restricted cytotoxic T lymphocytes (CTL), rapidly succumb to MTB infection 8.

The only currently available vaccine against TB is the Bacillus Calmette‐Guérin (BCG) vaccine. The BCG vaccine is routinely administered to protect children against severe TB but fails to protect against pulmonary TB in adults and has safety issues in HIV‐positive or otherwise immunocompromised individuals 9, 10. Efforts have been made to identify immunogenic MTB antigens that could be used in a subunit vaccine to boost immune responses to MTB infection 11, 12, 13, 14. Identification of CD8^+^ T cell epitopes associated with protective responses would provide insight for the design of more effective vaccines against TB 15.

Epitopes recognized by the CD8^+^ T cell receptor are presented by HLA class I molecules expressed on the surface of antigen‐presenting cells, resulting in CD8^+^ T cell activation. Several MTB epitopes have been identified that are presented by HLA class I molecules to T cells leading to restricted infection. For instance, many epitopes derived from antigens like ESAT‐6, Ag85B and PPE68 were found to be immunogenic 16, 17, 18. However, these analyses were limited either to a few MHC alleles (primarily HLA‐A2) or a confined subset of candidate antigen proteins.

In this study, we sought to identify novel CD8^+^ T epitopes derived from MTB proteins that could be presented by the MHC class I molecule, HLA‐A*11:01. HLA‐A*11:01 is a predominant allele in Southeast Asia where MTB infections are common 19. Our hope is that the MHC class I‐restricted CD8^+^ T epitopes identified in this study contribute to the development of an effective subunit vaccine against MTB infection.

## Materials and methods

### Study subjects

This study was approved by the ethics committee of the Southern Medical University. HIV‐negative patients diagnosed with TB were recruited from the Guangzhou Chest Hospital after obtaining written informed consent. Healthy individuals were recruited from Southern Medical University. HLA class I genes were genotyped using sequence‐based typing at the Beijing Genomic Institute.

### Preparation of peripheral blood mononuclear cells

Peripheral blood mononuclear cells (PBMCs) were isolated from fresh whole blood by density gradient centrifugation using Hypaque‐Ficoll (GE Healthcare Bio‐sciences AB, Uppsala, Sweden). The isolated PBMCs were resuspended in a mixture of 90% foetal bovine serum (FBS) and 10% DMSO (Sigma‐Aldrich, St. Louis, MO, USA), and frozen in liquid nitrogen until use.

### Bioinformatic analysis

Ninety‐four MTB proteins were chosen as sources for potential HLA‐A*11:01 restricted peptide epitopes as guided by previous studies. Among them, 45 proteins were selected from an analysis undertaken by Zvi *et al*. designed to identify TB vaccine candidates (Table 1, Part 1) 12. Sixteen proteins were reported to significantly reduce MTB loads in mouse vaccination experiments (Table 1, Part 2) 11. The remaining 33 MTB proteins were representative of antigens reported to elicit substantial CD8^+^ T cell responses (Table 1, Part 3) 20, 21, 22, 23, 24, 25, 26, 27, 28, 29. Amino acid sequences of 94 MTB proteins were obtained from GenBank. The sequences were scanned for HLA‐A*11:01‐restricted epitopes using the *NetMHCcons* prediction method (www.cbs.dtu.dk/services/NetMHCcons) 30. Epitopes were ranked according to their predicted binding affinity to HLA‐A*11:01, which was reported as a 50% inhibitory concentration (IC_50_) and ranked as high (IC_50_ ≤50 nM), intermediate (50 nM < IC_50_ ≤ 500 nM) or weak (IC_50_ >500 nM) binders 31. Forty‐eight peptides with a predicted IC_50_ <15 nM were selected for further analysis. In addition, one peptide with an IC_50_ of 50–500 nM (intermediate binder) and another peptide with an IC_50_ >500 nM (weak binder) were used as control peptides. Epitopes were checked on the Immune Epitope Database to ensure they had not been reported previously 32. The epitopes were evaluated for homology to confirm their uniqueness within the MTB genome/proteome (http://www.ncbi.nlm.nih.gov:80/blast/).

**Table 1:** *Mycobacterium tuberculosis* antigens (*n* = 94) selected for HLA‐A*11:01‐restricted epitope mapping

Part 1*			Part 2†	Part 3‡	
Rv0079	Rv1908c	Rv2629	Rv0496	Rv0315	Rv2034
Rv0288	Rv1926c	Rv2744c	Rv0577	Rv0350	Rv2108
Rv0467	Rv1980c	Rv2780	Rv0733	Rv0440	Rv2244
Rv0685	Rv1996	Rv3127	Rv0831	Rv0475	Rv2324
Rv0824c	Rv2005c	Rv3130c	Rv1411	Rv0754	Rv2380c
Rv0867c	Rv2006	Rv3131	Rv1569	Rv0932c	Rv2468c
Rv1009	Rv2029c	Rv3132c	Rv1626	Rv0934	Rv2770c
Rv1130	Rv2030c	Rv3347c	Rv1789	Rv0978c	Rv2903c
Rv1169c	Rv2031c	Rv3804c	Rv1860	Rv1363c	Rv3353c
Rv1174c	Rv2032	Rv3873	Rv2220	Rv1478	Rv3418c
Rv1349	Rv2389c	Rv3875	Rv2608	Rv1787	Rv3420c
Rv1733c	Rv2450c		Rv2875	Rv1788	Rv3615c
Rv1738	Rv2620c		Rv3020c	Rv1790	Rv3716c
Rv1793	Rv2623		Rv3044	Rv1791	Rv3803c
Rv1813c	Rv2626c		Rv3478	Rv1837c	Rv3874
Rv1884c	Rv2627c		Rv3619c	Rv1956	Rv3914
Rv1886c	Rv2628			Rv1966	

*Reference 12; ^†^Reference 11; ^‡^Reference 20, 21, 22, 23, 24, 25, 26, 27, 28, 29.

### Peptide competition binding assay

The binding affinity of peptides to HLA‐A*11:01 was assessed using the UV‐induced peptide exchange assay 33. Briefly, 50 candidate peptides (see above) were synthesized with a purity of more than 75%. Conditional HLA‐A*11:01 complexes containing UV‐labile peptide ligands were exposed to UV light (366 nm) in the presence or absence of candidate peptides for 30 min. The efficiency of peptide exchange was assessed using an HLA class I‐specific ELISA detecting the β2m, which signals successful HLA class I complex formation. The ELISA product is monitored *via* absorbance at 414 nm. The net average absorbance of each candidate peptide was normalized to that of the positive control peptide (EBNA_416‐424_: IVTDFSVIK) 34. The negative control peptide (NPKASLLSL) was included to confirm the specificity of the assay.

### Peptide synthesis

Based on the results of UV‐induced peptide exchange assay, the highest binding epitopes were selected for synthesis. These peptides were synthesized by Proimmune (Oxford, UK) and had a purity ≥96% as assessed by HPLC and mass spectroscopy. Peptides were dissolved in PBS or distilled water containing 10% DMSO to stock concentrations of 1 mg/ml. All peptides were aliquoted and stored at −20°C until use.

### IFN‐γ ELISPOT assays

ELISPOT assays were performed according to the manufacturer's instructions to test the ability of the candidate epitopes to activate cytokine release (Mabtech AB, Ellensviksvagen, Sweden). Briefly, PBMCs were thawed and rested overnight before use. Dead cells were removed using magnetic beads (Miltenyi Biotec, Bergisch Gladbach, Germany) to ensure viability. Peripheral blood mononuclear cells were seeded (2.5 × 10^5^ cells/well) in anti‐IFN‐γ‐coated ELISPOT plates in 100 μl of complete medium (RPMI + 10% FBS) containing 10 μg/ml peptide. Cells stimulated with phytohemagglutinin (PHA) were used as a positive control and cells cultured in medium alone were used as negative controls. We included a reported HLA class I A*11:01‐restricted epitope peptide (Rv0288_3‐11_, QIMYNYPAM) 35 and ESAT‐6 as referenced in this assay. After incubation for 24 hrs, plates were processed on an ImmunoSpot Analyzer S5 Versa (Cellular Technology Ltd., Cleveland, OH, USA) and spots were counted by ImmunoSpot software 5.0.3 (Cellular Technology Ltd). Results are expressed as the mean number of spot‐forming cells (SFC) per 10^6^ cells from triplicate assays.

Only assays with <50 spots/10^6^ cells for the negative control and >300 spots/10^6^ cells for the PHA positive control were considered valid 36. A response was defined as positive if two criteria were met: (*i*) the SFC per million of PBMC at least twofold over background, and (*ii*) the SFC per million of PBMC >50 spots.

### T cell proliferation assays

T cell proliferation was assessed using carboxyfluorescein succinimidyl ester (CFSE) assays as described previously 37, 38. Briefly, PBMCs from study subjects were thawed and dead cells were removed using magnetic beads (see above). The viable cells were washed with PBS and labelled with CFSE (Molecular Probes, Leiden, the Netherlands) at a final concentration of 5 μM for 10 min. at room temperature. After washing and counting, viable cells were seeded in 96‐well round‐bottom plates at a concentration of 2 × 10^5^ cells/well in the presence or absence of each peptide (10 μg/ml) 38 and incubated for 7 days. Phytohemagglutinin (10 μg/ml) was used as positive control to stimulate T cells.

On day 7, cells were stained 20 min. at 4°C for surface markers with anti‐CD8‐phycoerythrin (PE; eBioscience, San Diego, CA, USA) and anti‐CD3‐PE‐Cy 7 (eBioscience) and analysed by flow cytometry. Gated CD8^+^ T cells were examined for CFSE incorporation in proliferated CD8^+^ T cells. The proportion of proliferating CD8^+^ T cells was determined based on the reduction in CFSE fluorescence over time.

### Dextramer staining of MTB specific T cells

An HLA‐A*11:01 allophycocyanin (APC)‐labelled dextramer carrying Rv3130c_194‐204_ (p12) and an HLA‐A*02:01 APC‐labelled dextramer carrying an irrelevant epitope, ENV_120‐128_ (derived from HIV‐1 envelope glycoprotein gp160) 39, were purchased from Immudex (Copenhagen, Denmark). MHC‐dextramer staining was performed following the protocol offered by manufacturer. Briefly, PBMCs of active pulmonary TB patients were stained with dextramers for 10 min. at room temperature, followed by incubation with anti‐CD3 and anti‐CD8 fluorescence‐conjugated mAbs (eBioscience) for 20 min. Then, the cells were analysed by flow cytometry.

### Statistical analysis

Data are expressed as mean ± S.D. Comparisons between different groups were analysed using anova followed by Tukey's post test correction. Differences with a value of *P* < 0.05 were considered statistically significant. Data were analysed using GraphPad Prism 5.0 software (GraphPad Software Inc., San Diego, CA, USA).

## Results

### Prediction of epitopes

**Figure 1:**
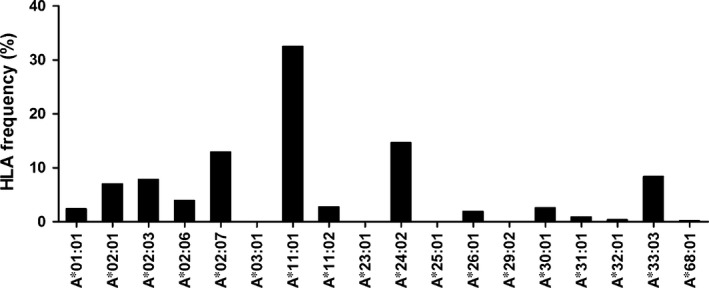
Frequency of HLA‐A alleles in active pulmonary TB patients from southern China. Fresh blood samples of active pulmonary TB patients (*n* = 350) were collected and HLA alleles were typed by the sequence‐based typing method. HLA‐A*11:01 is the most frequent allele in the HLA‐A loci as shown.

We tested 350 TB patients from southern China and discovered that HLA‐A*11:01 (32.5%) was the most frequent HLA‐A allele, with a frequency at least 10% higher than other alleles (Fig. 1). This was consistent with the reports published on Allele Frequency Net Database on HLA frequency distribution in China 40.

HLA‐A*11:01‐restricted CD8^+^ T cell epitopes were predicted by *NetMHCcons*. We choose 48 top‐ranked epitopes based on binding affinity. These epitopes consist of 8–11 amino acids, none of which had been defined as an epitope previously. Besides, we analysed the homology of the epitopes within the MTB genome/proteome and the uniqueness of their amino acid sequences were confirmed except for Rv1787_163‐172_ (p45), which has 100% sequence identity with Rv1790_163‐172_ (not included). Because both antigens are PPE family proteins with high identity among them, we did not exclude this epitope. The information of the epitopes is shown in Table 2. For control purposes, one peptide with an intermediate affinity (p49) and one with weak affinity (p50) were included.

**Table 2:** Predicted CD8^+^ T cell epitopes based on their affinity to HLA‐A*11:01

No.	Antigen	Sequence	Binding affinity (IC50, nM)^a^
p1	Rv3478_258‐267_	MTNTLHSMLK	4.67
p2	Rv2220258‐265	KTVTFMPK	5.29
p3	Rv3130c5‐13	TTLDAGFLK	5.90
p4	Rv1569_231‐239_	VVMTTTLSK	6.16
p5	Rv2029c186‐194	ISSGVFLLK	6.40
p6	Rv391478‐87	VSIPTLILFK	6.97
p7	Rv1884c	SSMTRIAK	7.16
p8	Rv119652‐62	MLFSMHGELYK	7.36
p9	Rv100934‐42	TAMRVTTMK	7.69
p10	Rv049642‐49	STIDEFAK	7.73
p11	Rv3347c1794‐1801	TTSPIPLK	7.94
p12	Rv3130c194‐204	AVMAGIVRAAK	8.07
p13	Rv1349477‐485	VSIARALLK	8.11
p14	Rv1790286‐295	AVAAPAFAEK	8.16
p15	Rv1980c122‐130	GTHPTTTYK	8.25
p16	Rv0867c9‐17	TTSNVSVAK	8.25
p17	Rv3873159‐166	AVNTLFEK	8.34
p18	Rv313182‐90	KVNRFPDPK	8.34
p19	Rv3347c2924‐2933	MTSGSSSGFK	8.34
p20	Rv1980c108‐115	GTQAVVLK	8.52
p21	Rv20061135‐1143	SVNNYQASK	8.61
p22	Rv0467326‐334	ATIAKFQK	8.80
p23	Rv1363c153‐161	MTSLDFNK	8.80
p24	Rv0824262‐269	VVMPVLKK	8.94
p25	Rv0440267‐275	GTFKSVAVK	9.65
p26	Rv3130c439‐448	AVARLVAISK	9.86
p27	Rv068526‐34	TTLTAAITK	9.86
p28	Rv1908c632‐639	RVLGANYK	10.07
p29	Rv232421‐29	ATFAEIGHK	10.69
p30	Rv1908c179‐187	KTFGFGFGR	11.11
p31	Rv2389c111‐120	KTQGPGAWPK	11.60
p32	Rv1980c26‐33	KTYCEELK	11.66
p33	Rv1411216‐223	SVQMTLSK	11.79
p34	Rv2108186‐196	TVFDYHNENAK	11.79
p35	Rv3132c501‐509	VSNAVRHAK	12.24
p36	Rv2780297‐307	CVANMPASVPK	12.58
p37	Rv2108114‐121	RVQTTVLK	12.64
p38	Rv2780250‐259	VSNSLVAHMK	12.64
p39	Rv2380c1276‐1283	AVAVVLHK	12.99
p40	Rv203237‐45	HTVALFLDK	12.99
p41	Rv195618‐26	AIAADPSFK	13.06
p42	Rv2030c289‐297	MTQWLIEEK	13.13
p43	Rv0932c192‐199	GTSDNFQK	13.20
p44	Rv1130239‐248	KAFAPAHAGK	13.71
p45	Rv1787163‐172	RLIPFAAPPK	13.71
p46	Rv2380c720‐728	TVINASRFK	13.79
p47	Rv0440261‐270	VVNKIRGTFK	13.94
p48	Rv1966108‐115	ATTAFGAK	14.09
p49	Rv1037c67‐74	QANAHGQK	326.55
p50	Rv1037c80‐89	NNMAQTDSAV	29,745.00

a Epitope‐binding affinity is indicated as 50% inhibitory concentration (IC50). IC50 ≤ 50 nM: high affinity; 50 nM ≤ IC50 ≤ 500 nM: intermediate affinity; IC50 ≥ 500 nM: weak affinity.

### Binding affinity of candidate peptides

**Figure 2:**
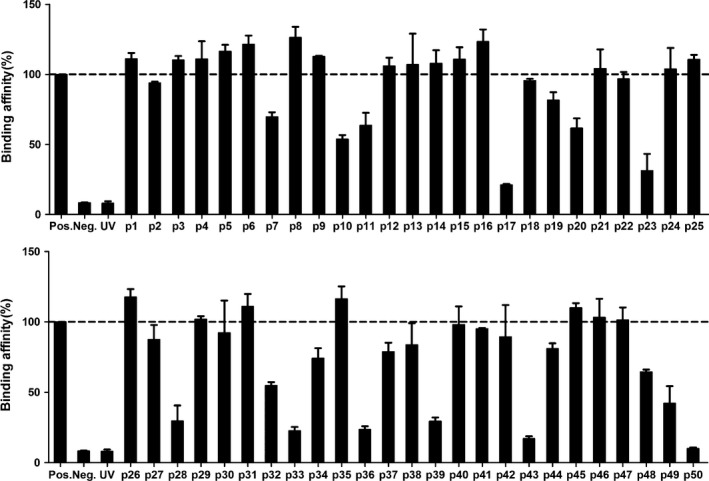
Binding affinity of predicted peptides to HLA‐A*11:01 by UV‐induced peptide exchange assay. HLA class I‐specific ELISA assays based on the detection of β2‐microglobulin were used to compare the stability of HLA complexes after UV‐illumination without (UV) or with rescue peptides (Pos., Neg. or predicted peptides). The net average absorbance of the exchanged peptide is normalized to Pos. Binding affinity of each peptide to HLA‐A*11:01 is reported as a percentage relative to Pos. The dotted line indicates a 100% cut‐off value. Bars represent mean ± S.D. of *n* = 3 for each group. Pos., positive control peptide; Neg., negative control peptide; UV, no peptide.

To evaluate the actual binding of candidate peptides to HLA‐A*11:01, we performed HLA stabilization assays utilizing UV‐induced peptide exchange. The binding of each peptide to HLA‐A*11:01 is reported as a percentage relative to the positive control peptide EBNA_416‐424_, which is a strong binder to HLA‐A*11:01. From these experiments, 22 peptides showed stronger binding (>100%) to HLA‐A*11:01 than the positive peptide; 11 peptides showed a relative binding of 81–98%, 16 peptides showed a relative binding of 17–79% and one peptide did not bind (Fig. 2). We set a cut‐off value on 100% for binders. A subset of 22 peptides >100% relative binding were selected for further studies to assess their ability to stimulate PBMCs.

### T cell recognition of peptides in active pulmonary TB patients

**Figure 3:**
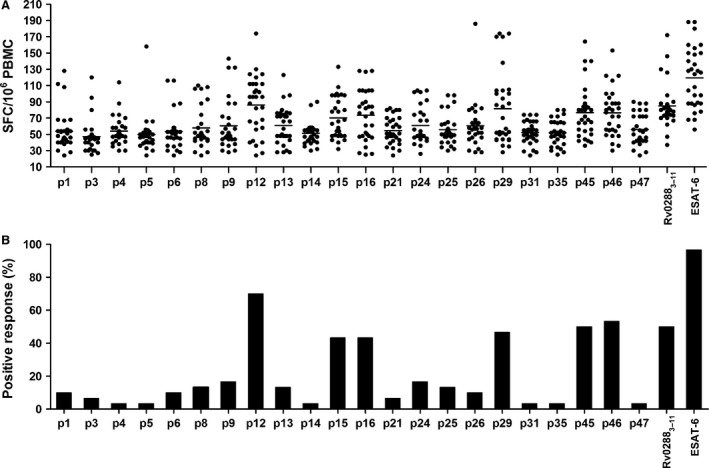
Recognition of HLA‐A*11:01‐restricted peptides by active pulmonary TB patients. PBMCs from HLA‐A*11:01‐positive active pulmonary TB patients (*n* = 30) were stimulated *in vitro* with individual peptides for 24 hrs. The number of epitope‐specific IFN‐γ‐producing T cells was determined by the ELISPOT assay (**A**). Percentage of HLA‐A*11:01‐positive active pulmonary TB patients responding to each individual epitope (**B**).

To examine the recognition of peptides in MTB‐infected subjects, we tested IFN‐γ response of PBMCs from 30 HLA‐A*11:01‐restricted active pulmonary TB patients. We used fresh peripheral blood‐derived PBMCs *ex vivo* to minimize artifacts from *in vitro* restimulation. The frequency of epitope‐specific IFN‐γ‐producing CD8^+^ T cells was determined by ELISPOT assay. Rv0288_3‐11_, a strong CD8^+^ T cell epitope belonging to the low molecular weight protein antigen 7 esxH (*i.e*., protein TB10.4) and antigen ESAT‐6, was used as positive control. Each of the 22 tested peptides was recognized by PBMCs of at least one patient. Moreover, all patients responded to at least one peptide, though some patients responded to as many as 9 or 10 peptides (*i.e*. patients 3 and 30) (Table 3). Six of the 22 tested peptides showed significant recognition by active TB patients, with a rate of response of more than 40%. These six epitopes were derived from different proteins (Fig. 3B and Table 4). As expected, the antigen ESAT‐6 was recognized by almost all of the patients (29/30). The highest frequency of IFN‐γ‐producing CD8^+^ T cell responses was detected against p12 (Rv3130c_194‐204_) peptide epitope (70%), with a higher rate than that observed for Rv0288_3‐11_ (55%). Obviously, peptides that were widely recognized also elicited more potent T cell responses (Fig. 3A). Six epitopes that were extensively recognized by active TB patients were selected for further immunogenic analyses.

**Table 3:** Characteristics of patients with TB and recognized peptides

Patient ID	Gender	Age	HLA‐A*11:01 peptide recognition
1	M	25	p16/p24/P45/p46
2	M	30	p6/p9/p12/p15/p25/p29/46
3	F	35	p8/p9/p12/p16/p24/p25/p26/p29/p45/p46
4	M	33	p12/p24/p46
5	F	32	p3/p12/p35/P45
6	F	23	p1/p5/p9/p12/p13/p29
7	M	45	p12/p15/p21/p31/p45
8	M	39	p4/p15/p26/p29/45
9	M	29	p1/p3/p6/p8/p14/p21/p46
10	F	29	p1/p12/p15/p29/p46
11	F	68	p15/p16/p45/p46
12	M	22	p13/p15/p45
13	F	21	p12/p15/p29/p47
14	F	25	p8/p13/p16/p29
15	F	26	p12/p13/p15/p16/p26
16	F	28	p15/45/p46
17	M	43	p12/p16
18	M	40	P12/p16/p29
19	F	40	p12/p45
20	M	44	p12/p16/p46
21	M	21	p45
22	M	32	p12/p15/p46
23	M	38	p12/p16/p29
24	F	35	p12/p15/p45
25	F	40	p12/p16/p29/p46
26	F	50	p16/24/p45/p46
27	M	43	p6/p9/p12/p15/p25/p29/p46
28	M	47	p12/p15/p16/p29/p45/p46
29	M	31	p12/p16/p29/p45
30	F	26	p8/p9/p12/p16/p24/p25/p29/p45/p46

**Table 4:** Information of six MTB proteins^a^

Epitope	Gene name	Molecular mass (kD)	Product
Rv3130c194‐204	Tgs1	50.7	Triacylglycerol synthase Tgs1
Rv1980c122‐130	Mpt64	24.8	Immunogenic protein Mpt64
Rv0867c9‐17	RpfA	39.9	Resuscitation‐promoting factor RpfA
Rv232421‐29	Rv2324	16.3	Transcriptional regulatory protein
Rv1787163‐172	PPE25	37.1	PPE family protein PPE25
Rv2380c720‐728	MbtE	18.3	Peptide synthetase MbtE

a Information is from http://www.tbdb.org/.

**Figure 4:**
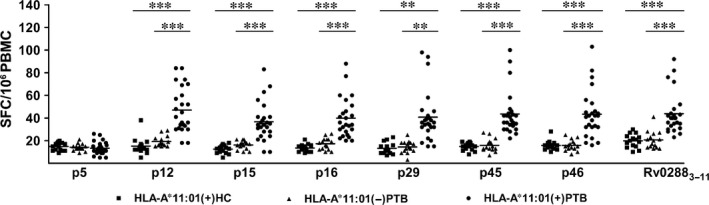
Comparison of the frequency of epitope‐specific IFN‐γ‐producing CD8^+^ T cells in HLA‐A*11:01‐positive healthy individuals, HLA‐A*11:01‐negative active pulmonary TB patients and HLA‐A*11:01‐positive active pulmonary TB patients. PBMCs from healthy individuals (*n* = 15) and active pulmonary TB patients (*n* = 15 and *n* = 25 for HLA*11:01‐negative and ‐positive individuals, respectively) were stimulated *in vitro* with individual peptides for 24 hrs and the frequency of epitope‐specific IFN‐γ‐producing CD8^+^ T cells were determined by the ELISPOT assay. *P < 0.05, **P < 0.01, ***P < 0.001 versus the HLA‐A*11:01(+)PTB.

Next, we compared the IFN‐γ‐producing CD8^+^ T cells from HLA‐A*11:01‐positive healthy individuals and HLA‐A*11:01‐negative active pulmonary TB patients with that of HLA‐A*11:01‐positive patients. For all six epitopes, significantly (*P* < 0.05) more CD8^+^ T cells from HLA‐A*11:01‐positive TB patients produced IFN‐γ than cells from the other groups (Fig. 4). p5 (Rv2029c_186‐194_), which was inefficient at stimulating IFN‐γ production from the CD8^+^ T cells of HLA‐A*11:01‐positive patients (3% response), also failed to elicit significant IFN‐γ‐producing CD8^+^ T cell responses in other two groups (Fig. 4).

### Specific CD8^+^ T cell proliferation induced by HLA‐A*11:01‐restricted peptides

The epitopes were further assessed for their capacity to induce CD8^+^ T cell proliferation in active TB patients and healthy subjects. Peripheral blood mononuclear cells were labelled with CFSE and restimulated *in vitro* with individual epitope peptides.

**Figure 5:**
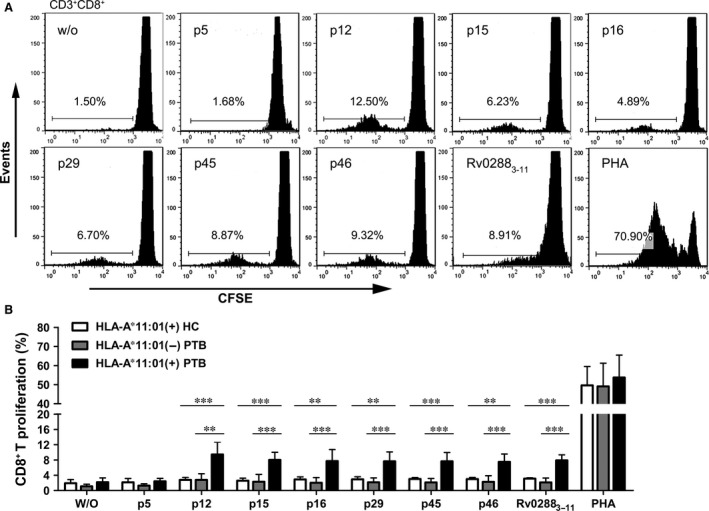
Epitope‐specific CD8^+^ T cell proliferation in PBMCs from active pulmonary TB patients. PBMCs from HLA‐A*11:01‐positive healthy individuals, HLA‐A*11:01‐negative active TB patients and HLA‐A*11:01‐positive active pulmonary TB patients were stimulated with individual peptides or PHA. After 7 days, CD8^+^ T cell proliferation were examined by flow cytometry. Plots are gated on CD3^+^ CD8^+^. The proliferation of CD8^+^ T cells is shown as the percentage of decrease in CFSE expression. (**A**) A proliferation profile of CD8^+^ T cells from a representative active pulmonary TB patient after *in vitro* stimulation with individual epitopes for 7 days. (**B**) Proliferation of epitope‐specific CD8^+^ T cells in HLA‐A*11:01‐positive healthy individuals, HLA‐A*11:01‐negative active TB patients and HLA‐A*11:01‐positive active TB patients. Bars represent mean ± S.D. of *n* = 10 for each group. w/o: negative control; *P < 0.05, **P < 0.01, ***P < 0.001 versus the HLA‐A*11:01(+)PTB.

Figure 5A shows representative proliferation profiles of CD8^+^ T cells (one patient) in response to different epitopes and control conditions in one active TB patient. Following stimulation of PBMCs with epitopes or PHA (positive control), significant CD8^+^ T cell proliferation was observed. Little proliferation was observed in response to peptide Rv2029c_186‐194_ or medium alone. We also tested the proliferation of CD8^+^ T cells from HLA‐A*11:01‐positive healthy individuals (*n* = 10) and HLA‐A*11:01‐negative TB patients (*n* = 10) after *in vitro* restimulation with different epitopes. As expected, epitope‐specific CD8^+^ T cell expansion only occurred in in PBMCs from HLA‐A*11:01‐positive TB patients (*n* = 10) (Fig. 5B).

### HLA‐A*11:01 tetramer staining of epitope specific CD8^+^ T in active TB

**Figure 6:**
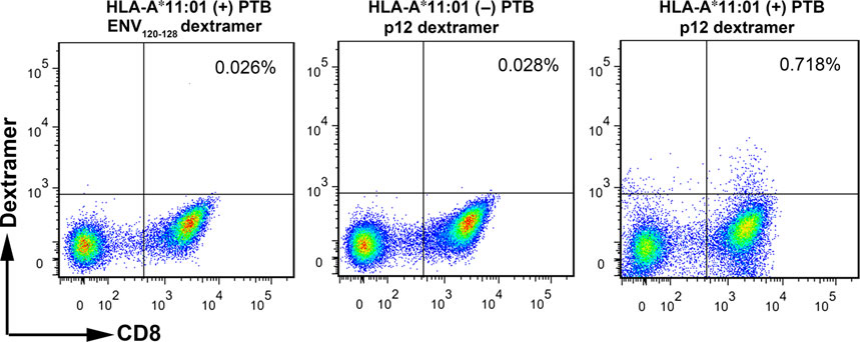
Dextramer staining of epitope‐specific CD8^+^ T cell in active pulmonary TB patients. PBMCs from HLA‐A*11:01‐positive pulmonary TB patients (*n* = 4) were stained with dextramers displaying epitopes ENV_120‐128_ (left panel) or p12 (right panel), and PBMCs from HLA‐A*11:01‐negative active pulmonary TB patients (*n* = 4) were stained with dextramers displaying p12 (middle panel). PBMCs were costained with anti‐CD3 and ‐CD8 antibodies. Peptide‐specific CD8^+^ T cells were analysed by flow cytometry. Dextramers displaying ENV_120‐128_ and HLA‐A*11:01‐negative PBMCs were used as negative controls. Representative flow cytometry patterns are shown.

An HLA‐A*11:01 dextramer was constructed carrying the epitope p12, the epitope most frequently recognized by patient CD8^+^ T cells (Fig. 3B); an HLA‐A*02:01 dextramer carrying an irrelevant peptide (ENV_120‐128_) was used as negative control. Peripheral blood mononuclear cells from HLA‐A*11:01‐positive or ‐negative TB patients were stained *ex vivo* with optimized concentrations of APC‐labelled dextramers. While significant amount of epitope‐specific CD8^+^ T cells were detectable in PBMCs from HLA‐A*11:01‐positive TB patients (Fig. 6, right panel), no dextramer‐positive CD8^+^ T cells were observed in the control groups (Fig. 6, left and middle panels). This demonstrates that MTB‐specific CD8^+^ T cells accumulate to a substantial number in response to exposure to p12.

## Discussion

Tuberculosis remains a major public health problem and economic burden worldwide because of the morbidity and mortality caused by this disease 41. Intense efforts had been offered to develop a new vaccine to prevent this deadly disease. Mouse studies have suggested that MHC class I‐mediated CD8^+^ T cells have a critical role in protective immunity against TB 42. Identification of antigens and/or their epitopes that are capable of boosting CD8^+^ T cell responses could be of great importance in vaccine development.

Although new immunogenic MTB antigen epitopes have been defined recently, significant voids remain in the area of epitope identification. Epitopes detectable by human T cells have only been identified in approximately 4% of the MTB proteome and about 65% of the reported epitopes are derived from the top 30 most frequently studied proteins (*e.g*., ESAT‐6 and CFP‐10), leaving the majority of the MTB proteome (consisting of approximate 4000 proteins) unexplored. In addition, relatively few of the known epitopes reported are detectable by CD8^+^ T cell epitopes 32, 44. Present vaccine candidates are often based on a limited number of T cell protein or epitope antigens, which might narrow the breadth of the immune response. For example, focused immune response against immunodominant epitopes were reported to deregulate the immune system's ability to centre on protective subdominant epitopes of MTB 45, 46 and were associated with a poor prognosis in an AIDS model 47.

Furthermore, most published CD8^+^ T cell epitopes to date are presented by HLA subtypes expressed by Caucasian populations, particularly the HLA‐A*02:01 allele 40, 43. This is problematic because the highest TB burdens in the world are located in South‐East Asia, Sub‐Saharan Africa and Western Pacific 3. As more attention is put into these areas, we need to identify immunodominant epitopes restricted by HLA‐type prominent in these areas, such as HLA‐A*11:01, to develop new subunit vaccines and diagnostic strategies.

Binding affinity to allele‐specific MHC molecules proved to be an important parameter for discovering immunodominant epitopes. A study led by Sette built a binding threshold that was suitable for many known CTL epitopes 31. This finding was confirmed by a later study, which suggested the correlation between affinity and immunogenicity 48. An increasing number of *in silico* methods based on binding affinity have been developed. Algorithms that narrow the number of peptides that may generate peptide‐MHC interactions reduce both experimental and financial burdens 49. *NetMHCcons* is a consensus method for the MHC I binding motif predictions, which takes advantage of three mainstream prediction methods including *NetMHC*,* NetMHCpan* and *Pickpocket* 30. However, experimental assays are still needed to validate these predictions. The UV‐induced peptide exchange technique, introduced first by Ton N M Schumacher for the generation of MHC multimers, is well suited for the rapid assessment of MHC binding properties of extensive peptide libraries 33, 50. EBNA_416‐424_ (IVTDFSVIK) is an immunogenic epitope with high affinity to HLA‐A11 and properly used as a reference to narrow the scope and obtain optimal epitopes, although this reference method may miss some epitopes for which the binding affinity is below the cut‐off value.

Binding affinity alone is not sufficient for immunogenicity; functional assays are also needed to validate the predicted peptide epitopes. Various types of functional assays are used for detection of antigen‐specific T cells, such as ELISPOT, intracellular cytokine stain and cytokine capture. ELISPOT assay is sensitive for low‐frequency antigen‐specific T cells detection, with a resolution of one in 100,000 cells, which is one or two orders of magnitude higher than that of flow cytometry‐based techniques 51. In addition, ELISPOT assay identifies antigen‐specific T cells at the single cell level without long‐term *in vitro* culture. This provides the unbiased information of the *in vivo* cellular frequency in an individual and offers a strong evidence of immunological activation 51, 52. Carboxyfluorescein succinimidyl ester has been found to be effective at monitoring T cell division since its first description by Lyons and Parish, and then became a routine tool for the assessment of proliferation of lymphocytes with its high sensitivity and low toxicity to cells 37, 53. In our experiments, significant epitope‐specific CD8^+^ T cell proliferation was observed after 7 days of stimulation in active TB patients, which highlights the capability of our epitopes in boosting the MTB‐specific responses.

Only limited number of studies have reported the use of an MHC tetramer to gauge MTB‐specific T cells in PBMCs. In most cases, the HLA‐A*02:01 tetramer has been used. Multimers constructed with other HLA molecules are needed to visualize the complexity of MHC‐restricted T cell responses 54. Dextramers have a higher valency than tetramers and are useful for the detection of T cells with low‐affinity T cell receptors 55. In this study, we used a dextramer synthesized with the Rv3130c_194‐204_ epitope and found a significantly higher fraction of CD8^+^ T cells stained positive for the Rv3130c_194‐204_ dextramer from HLA‐A*11:01‐positive patients than from the controls. This further confirmed the HLA restriction of this epitope. Importantly, *ex vivo* identification of the epitope‐specific T cells interaction signifies the value of this particular epitope for vaccine development and clinical diagnosis. Because of our limited funds, we couldn't afford to construct dextramers loaded with the other five epitopes. However, it is highly probable that antigen‐specific CD8^+^ T cells could be detected for these other epitopes because they displayed immunogenic properties similar to Rv3130c_194‐204_.

In conclusion, combining algorithmic prediction with experimental validation, we defined six novel CD8^+^ T cell epitopes. These epitopes were derived from six different antigenic MTB proteins (Rv3130c, Rv1980c, Rv0867c, Rv2324, Rv1787 and Rv2380c) and are presented by HLA‐A*11:01. Epitope‐specific CD8^+^ T cells were detected by an HLA‐A*11:01 dextramer loaded with one of the six epitopes, Rv3130c_194‐204_. Our results suggested that these epitopes might serve as potential targets for future vaccine design and diagnostic strategies.

## Conflict of interest

The authors declare no conflicts of interest.
